# Study on symptom dimensions and clinical characteristics in patients with obsessive-compulsive disorder

**DOI:** 10.1590/1806-9282.20230676

**Published:** 2024-03-15

**Authors:** Xuan Liu, Yuehan Zhao, Pengchong Wang, Xiangyun Yang, Zhanjiang Li

**Affiliations:** 1 Capital Medical University, Beijing Anding Hospital, National Clinical Research Center for Mental Disorders and National Center for Mental Disorders, Beijing Key Laboratory of Mental Disorders – Beijing, China.; 2 Capital Medical University, Advanced Innovation Center for Human Brain Protection – Beijing, China.; 3 Weifang People's Hospital, Department of Clinical Psychology – Weifang, China.

**Keywords:** Obsessive-compulsive disorder (OCD), Symptom, Cross-sectional studies, Cluster analysis

## Abstract

**BACKGROUND AND OBJECTIVE:**

The aim of this study was to explore the symptom dimensions and clinical characteristics of obsessive-compulsive disorder in the context of Chinese culture.

**METHODS:**

In this cross-sectional study, the severity of obsessive-compulsive symptoms, the distribution of symptoms, and symptom scores of 263 patients with obsessive-compulsive disorder were assessed using the Yale-Brown Obsessive-Compulsive Scale and Yale-Brown Obsessive-Compulsive Inventory Symptoms Checklist. System cluster analysis and Pearson analysis were performed to explore the relationships between the main clinical characteristics and symptom dimensions.

**RESULTS:**

Cluster analysis identified four symptom dimensions of obsessive-compulsive disorder: (1) symmetry precision; (2) contamination cleaning; (3) aggression examination; and (4) taboo thinking. The symmetry precision dimension showed an association with years of education. The compulsive score, total Yale-Brown Obsessive Compulsive Scale score, contamination cleaning dimension, and aggression examination dimension had significant relationships. Age, age at onset, obsessive score, and compulsive score had a significant correlation with the taboo-thinking dimension.

**CONCLUSION:**

The symptom dimensions of obsessive-compulsive disorder in China are similar to those in other regions. Each of the four symptom dimensions had distinct clinical characteristics.

## INTRODUCTION

Obsessive-compulsive disorder (OCD) is a common psychiatric disorder characterized by obsessions (recurrent intrusive thoughts with excessive anxiety) and compulsions (excessive repetitive actions used to reduce obsession-induced anxiety)^1,2^. Approximately 3% of the world's population is affected by OCD^3^, resulting in high social and economic burden^4^. Increasing evidence has suggested that OCD is an extremely heterogeneous mental disorder^5^. Patients with the same definite diagnosis of OCD may have very different clinical manifestations^6^, which may be related to different genetic and neurobiological mechanisms, resulting in different onset characteristics, manifestations, treatment methods, effects, and prognoses^7^. This not only affects our ability to explore the pathogenesis of OCD but also presents challenges in selecting effective treatment options for patients^8^.

In clinical practice, most patients with OCD often exhibit both obsessive thinking and compulsive actions^9^. The classification method in the International Classification of Diseases (ICD) 10th revision (ICD-10) cannot be used to select effective clinical treatment plans and has been removed from ICD-11. Thus, there is currently no unified clinical classification or evaluation standard for OCD. Although many studies have explored the symptom dimensions of OCD and attempted to lay a foundation for its classification, no conclusions have been reached.

This study aimed to discuss the symptom dimensions of OCD in China. We attempted to explore the symptom dimensions of Chinese patients with OCD through a systematic cluster analysis of the categories of the Yale-Brown Obsessive-Compulsive Scale Checklist (Y-BOCS-CL), compare the results obtained with those of a previous study, and explore the relationships between the main clinical characteristics of patients and the symptom dimensions obtained in our study.

## METHODS

### Participants

A total of 263 outpatients with OCD were recruited from Beijing Anding Hospital, Capital Medical University, and Weifang People's Hospital between September 2017 and September 2021 who met the diagnostic criteria outlined in the Diagnostic and Statistical Manual of Mental Disorders, fourth edition (DSM-4)^10^. Diagnosis was made by attending psychiatrists with significant experience in diagnostic interviews using the Mini International Neuropsychiatric Interview (MINI)^11^. The severity of illness was determined using the Y-BOCS^12^. Patients were included if they were between the ages of 18 and 65 years, had a score ≥7 on the Y-BOCS, and had a cultural level of junior high school or above. Exclusion criteria included schizophrenia, bipolar disorder, mental retardation, OCD occurring exclusively in the context of depression, a history of organic brain disease, major physical disease, drug dependence, and psychoactive substance use. This study was approved by the Research Ethics Committee of Beijing Anding Hospital, Capital Medical University (201941FS-2).

All participant symptoms were assessed using the Y-BOCS and Y-BOCS SC. The Y-BOCS was used to evaluate the severity of obsessive-compulsive symptoms and has satisfactory interrater reliability and construct validity. The Y-BOCS includes the obsessive, compulsive, and total scale scores, with a higher score indicating more severe symptoms. A total Y-BOCS score of <16 is classified as mild or subclinical; 16–22 is classified as moderate; 23–31 is classified as severe; and >31 is classified as extremely severe^13,14^.

The Y-BOCS SC is a semistructured interview outline for the Y-BOCS, which comprises eight categories of obsessions (aggressive, contamination, sexual, hoarding/saving, religious, symmetry or exactness, somatic, and miscellaneous) and seven categories of compulsions (cleaning/washing, checking, repeating, counting, ordering/arranging, hoarding/saving, and miscellaneous). With a total of 68 items, the Y-BOCS SC has been extensively used in research and clinical settings for the past two decades and is generally assumed to possess good reliability and validity^15^. However, the two categories related to hoarding, each containing two items, were not evaluated, as hoarding disorder is regarded as an independent diagnosis of OCD in the DSM-5^16^. Likewise, two miscellaneous categories, which encompass 17 items and exhibit high heterogeneity and low mutual consistency, were excluded from this study. As different patients exhibit different manifestations, it is not feasible to conduct unified data processing. Consequently, this study eliminated the two hoarding categories and two miscellaneous categories of the Y-BOCS, leaving 11 categories to process the data.

### Statistical analysis

All collected data were entered into the SPSS software version 26.0 for Windows (IBM/SPSS Inc., New York, USA). Pearson analysis was conducted to explore the relationships between the main clinical characteristics and symptom dimension scores in our sample. Statistical significance was assumed at p<0.05.

## RESULTS

### Demographic and clinical findings

In total, 263 patients were included in the study, comprising 142 males (54.0%) and 121 females (46.0%). The average age of the participants was 32.09±8.32 years (18–64 years), with a mean age of onset of 21.94±6.81 years. The duration of the illness ranged from 1 month to 37 years, with an average of 6.76±6.63 years.

Approximately 72.62% (191/263) of patients were treated with serotonin reuptake inhibitors (SRIs). The use of benzodiazepines, such as diazepam, lorazepam, and oxazepam, was uncommon (36/263, 13.69%). A total of 10.27% (27/263) of participants received low doses of atypical antipsychotics, including risperidone, olanzapine, aripiprazole, and quetiapine. Cognitive-behavior therapy (CBT) was administered to a small proportion of the participants (33/263, 12.55%). Detailed clinical and demographic data are presented in Table 1.

**Table 1:** Demographic and clinical characteristics of participants.

Clinical variables	OCD (n=263) Mean±SD/frequency
Age (years)	32.09±8.32
Gender (male/female)	142/121
Marital status (single/married)	141/122
Years of education	14.20±2.79
Ethnicity (Han/other)	246/17
Religion (yes/no)	20/243
Family history (negative/positive)	228/35
Age at onset (years)	21.94±6.81
Duration of illness (years)	6.76±6.63
Y-BOCS total score	22.17±6.47
Obsession score	11.49±4.55
Compulsion score	10.68±4.89
Currently on SRI (yes/no)	191/72
Any benzodiazepine (yes/no)	36/227
Any antipsychotic (yes/no)	27/236
On CBT (yes/no)	33/230

OCD: obsessive-compulsive disorder; Y-BOCS: Yale-Brown Obsessive Compulsive Scale; SRI: serotonin reuptake inhibitors; CBT: cognitive-behavior therapy.

### System cluster analysis findings

**Figure 1:**
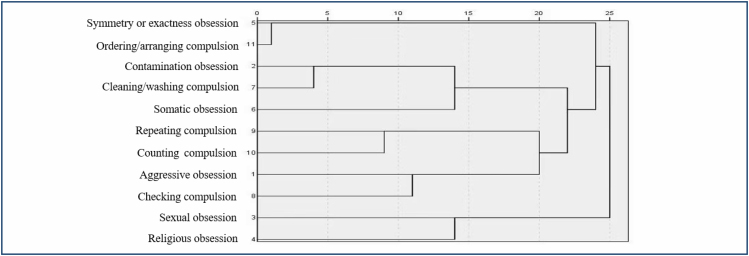
System cluster analysis results of the 11 Yale-Brown Obsessive-Compulsive Scale Symptoms Checklist categories.

Figure 1 presents the cluster analysis outcomes after scoring 11 Y-BOCS SC categories. The 11 Y-BOCS SC categories were divided into two, three, four, or five symptom dimensions. Combining domestic and foreign research results on OCD symptom content classification, this study surveyed 19 senior psychiatrists specialized in OCD to determine the content classification of OCD symptoms. Among the experts, 53% (10) supported the four-dimensional classification of OCD symptom content: symmetry and precision, contamination and cleanliness, aggressive examination, and taboo thinking. This study adopted four symptom dimensions (Figure 1).

### Findings from Pearson analysis

Pearson analysis revealed a significant relationship between years of education and Dimension 1 (symmetry precision dimension, r=-0.13) (Table 2). Additionally, there were significant relationships between compulsive score, total Y-BOCS score, and Dimension 2 (contamination cleaning dimension, r=0.19, and r=0.23, respectively) and Dimension 3 (aggression examination dimension, r=0.17, and r=0.17, respectively). Furthermore, age, age at onset, obsessive score, and compulsive score showed significant relationships with Dimension 4 (taboo-thinking dimension, r=-0.12, r=-0.12, r=0.25, and r=-0.16, respectively).

**Table 2:** Correlation between the main clinical characteristics and four symptom dimensions.

Variables	Dimension 1		Dimension 2		Dimension 3		Dimension 4	
	r	P	r	P	r	P	r	P
Gender	-0.04	0.57	0.08	0.22	-0.05	0.42	-0.05	0.45
Age	-0.08	0.21	<0.01	0.99	0.08	0.21	-0.12*	0.046
Age of onset	-0.04	0.57	0.07	0.25	0.01	0.83	-0.12*	0.044
Disease duration	-0.05	0.43	-0.10	0.10	0.05	0.39	-0.04	0.50
Years of education	-0.13*	0.04	<0.01	0.96	-0.09	0.17	-0.08	0.17
Family history	-0.07	0.24	-0.08	0.19	-0.01	0.86	-0.06	0.35
Obsessive score	0.07	0.24	0.11	0.09	0.06	0.37	0.25*	<0.01
Compulsive score	0.03	0.60	0.19*	<0.01	0.17*	<0.01	-0.16*	<0.01
Total Y-BOCS score	0.06	0.35	0.23*	<0.01	0.17*	<0.01	0.66	0.29

* p≤0.05 was considered to show a significant association. Dimension 1: symmetry precision dimension; Dimension 2: contamination cleaning dimension; Dimension 3: aggression examination dimension; and Dimension 4: taboo thinking dimension.

## DISCUSSION

In this study, system cluster analysis was used to analyze the symptoms of 263 patients with OCD. OCD symptoms in this study were divided into four dimensions: (1) symmetry precision dimension (this dimension included symmetry or exactness obsession and ordering/arranging compulsion); (2) contamination cleaning dimension (this dimension included contamination obsession, cleaning/washing compulsion, and somatic obsession); (3) aggression examination dimension (this dimension included repeating compulsion, counting compulsion, aggressive obsession, and checking compulsion); and (4) taboo thinking dimension (this dimension included sexual and religious obsessions).

Our study's findings were consistent with previous research, which identified the same main symptom dimensions in OCD. For instance, Pinto et al.^17^ and another study found five factors, namely, symmetry/ordering, hoarding, doubt/checking, contamination/cleaning, and taboo thoughts^17^. While our study excluded hoarding symptoms due to their separation from OCD as an independent diagnosis in DSM-5, the remaining dimensions were consistent. However, discrepancies exist between domestic and international studies, with classification methods ranging from 3 to 7. Although numerous studies have been conducted on OCD symptom dimensions, a definitive conclusion has not yet been reached.

Differing classification methods in OCD studies may be due to data processing techniques and symptom selection. Most studies used factor analysis, which may overlook some symptoms, while our study utilized system cluster analysis, which provides a more comprehensive understanding of symptom dimensions. Therefore, cluster analysis can lead to a more comprehensive understanding and analysis of the dimensions of obsessive-compulsive symptoms, a view that has also been confirmed by Cameron et al.^18^. Additionally, cultural and sample size differences may also contribute to discrepancies. Despite these differences, our study found consistent symptom dimensions in Western countries, suggesting stability across regions and sociocultural contexts.

The study found a significant correlation between years of education and Dimension 1, suggesting that patients with symmetry precision symptoms had fewer years of education. This aligns with previous studies that reported an association between symmetrical symptom groups and years of education^19^. Limited research is available on this phenomenon, indicating the need for further investigation.

The study found positive correlations between compulsive score, total Y-BOCS score, and Dimensions 2 and 3, indicating that patients with contamination or attack fears have more compulsions for repeated cleaning and examination. These findings align with another study that identified cleaning/washing, repeating/redoing, and checking as the most common types of compulsion^20^. Dimension 4 symptoms, primarily related to taboos surrounding sex, were more likely in younger individuals with earlier onset, higher obsessiveness, and lower compulsiveness. Lower religiosity in Chinese OCD patients may be linked to psychological factors in early adulthood.

This study had several limitations. First, the sample was drawn from only two locations in China, which may not be representative of the patients nationwide. Second, the coronavirus disease 2019 (COVID-19) pandemic may have affected the patients’ symptoms; thus, further research is necessary to confirm these findings. Finally, this cross-sectional study lacked follow-up data, which could potentially provide useful insights into the evolution of symptoms and validation of the current results.

## CONCLUSION

Our findings have revealed that the symptoms of OCD in Chinese patients are multi-dimensional. The four symptom dimensions identified in this study were consistent with those reported in previous studies, suggesting that OCD symptoms are similar across different regions. However, each dimension showed distinct clinical characteristics, which may indicate different pathogenic mechanisms underlying OCD. Our research provides a basis for future studies to explore the symptom dimensions, diagnosis, and pathogenesis of OCD.
